# Transition from pediatric to adult healthcare for patients with chronic functional constipation: a scoping review and provider insight

**DOI:** 10.1007/s00383-026-06537-w

**Published:** 2026-07-20

**Authors:** Emma J. Moore, Melissa Y. Tien, Susan M. Sawyer, Sebastian K. King, Misel Trajanovska

**Affiliations:** 1 Murdoch Children’s Research Institute, 50 Flemington Road, Parkville, Melbourne, VIC 3052 Australia; 2 Department of Paediatrics, University of Melbourne, 50 Flemington Road, Melbourne, VIC Australia; 3 Centre for Adolescent Health, The Royal Children’s Hospital, 50 Flemington Road, Melbourne, VIC Australia; 4 Department of Paediatric Surgery, The Royal Children’s Hospital, 50 Flemington Road, Melbourne, VIC Australia

**Keywords:** Transition, Transfer, Chronic constipation, Colorectal

## Abstract

Chronic functional constipation is complex and often requires a multifaceted treatment approach. Patients are treated by medical and surgical units in outpatient and inpatient settings. Some adolescents will require transition to adult services for ongoing management, but little is understood about the transitional care needs of this cohort. A scoping review of PubMed, MEDLINE and Embase examined transitional care for patients with chronic functional constipation aged 10 to 30 years. Additionally, correspondence was sought from hospitals with specialized Colorectal Centers. Three studies, all surgical in nature, met the inclusion criteria and 15 Colorectal Centers provided details of their management of patients with chronic functional constipation during the period of transition. There were no constipation-specific transitional care programs identified. Multiple departments in both pediatric and adult services managed patients with chronic functional constipation. Identified barriers and enablers aligned with general transitional care guidelines. Inconsistent approaches to treatment of chronic functional constipation made standardized transition to adult care a challenge. In essence, the transitional care needs of this cohort are largely unknown. These results indicate the value of further research to inform standardization of care, with the goal of improving patient outcomes during the critical transition from pediatric to adult services.

**Supplementary Information:**

The online version contains supplementary material available at 10.1007/s00383-026-06537-w.

## Introduction

While most people will experience constipation at some stage, others live with chronic constipation which can substantially reduce their quality of life [1]. There is significant variation in reported prevalence rates, with studies indicating that the worldwide prevalence is between 10 and 20% [1, 2]. Prevalence appears to increase with age, although this is difficult to accurately define [3]. It is believed that the prevalence of chronic constipation in children is between 9 and 14%, increasing to 16% in adulthood [4–6]. The most notable rise occurs later in adulthood with 33.5% of adults aged between 60 and 110 years affected [3]. Despite intervention in childhood, 40–50% of children with this condition will continue to have symptoms as they mature into adulthood [5, 7].

Chronic constipation is defined as constipation meeting the Rome IV criteria that does not resolve after one month in children or three months in adults [4, 8]. While some cases of constipation may be explained by the use of opioids, dietary, and lifestyle changes, or a structural deformity of the bowel, rectum or anus, in many cases no specific cause is identified [9]. For this study, chronic constipation where no cause is identified is referred to as chronic functional constipation. Various terms are used synonymously with chronic functional constipation, including chronic constipation, idiopathic constipation, functional constipation, and intractable constipation.

Patients with chronic constipation represent a challenge to the healthcare system. Unlike many conditions for which exploration and amplification of treatment options typically involves management within a single hospital department, patients with severe constipation can be managed by a variety of different medical and surgical units and multiple types of providers, even within the one institution.

Treatment may involve lifestyle modification, pharmaceutical intervention and, in severe cases, surgical intervention such as a colon resection, an appendicostomy, ileostomy or a colostomy [1, 9, 10]. Treatment is often based on a complex process of ‘trial and error’ to determine the most suitable bowel management plan [11]. The dynamic nature of the condition, and limited understanding of the role of motility in constipation, may also mean that treatment that worked one week does not work the next, which can result in this ‘trial and error’ approach continuing indefinitely [12–14].

The complexity of identifying and treating patients suggests that it is challenging to gain a true understanding of the size of this patient cohort and the extent of their ongoing healthcare needs, whether in primary care or specialist services. For example, Stephens and colleagues found that 40% of children admitted for constipation management had not received outpatient care prior to initial admission, possibly due to readily accessible over-the-counter medications. Re-admission rates were also notable with 10% of patients requiring re-admission within 12 months [15]. This complexity leads us to question what happens to those children who require ongoing management as they mature into adolescence and young adulthood. For the subset of adolescents with persisting symptoms related to constipation that reduces their quality of life, transition to adult healthcare is indicated. Transitional care programs have become increasingly prevalent, particularly for medical conditions such as Type 1 diabetes and cystic fibrosis; and there have been recent advances in transitional care for conditions that require surgical management, such as congenital cardiac disease [16–18].

We set out to review what is known about the care of adolescents with chronic functional constipation as they transfer to adult healthcare, including an analysis of the barriers to and enablers of successful transition, with the wider objective of informing future policies and programs.

## Methods

Using established nomenclature, we refer to ‘transfer of care’ as the point in time when healthcare formally shifts from the responsibility of the pediatric provider to the adult provider [19]. In contrast, we recognize ‘transition of care’ as a process that involves a gradual shift of responsibility from a carer (typically the parents) to the adolescent, that is facilitated by supporting the young person to become sufficiently empowered to manage their own care [19, 20]. Transfer may therefore be considered as a more specific component of the wider transition process, and akin to what graduation from high school is to continuing education, transition continues beyond the period of engagement with pediatric services [21].

### Study selection

This scoping study was conducted in accordance with the Preferred Reporting Items for Systematic Reviews and Meta-Analyses extension for Scoping Reviews (PRISMA-ScR) guidelines. A search of MEDLINE, Embase and PubMed was conducted on 8th June 2024 using the search strategy detailed in **Online Resource 1**. The search criteria were restricted to peer review articles published in English from January 2011 to June 2024 to ensure that the review captured contemporary practices. Editorials and letters were excluded. Articles were imported into Covidence [22] and two authors (EJM, MYT) independently screened titles, abstracts, and relevant full texts against the inclusion criteria. Citation searching was also performed but yielded no additional studies beyond those identified through the primary search. Our inclusion criteria were intentionally broad; we wished to identify research studies of people with chronic functional constipation, with a median age between 10 and 30 years, that focused on any aspect of transition or transfer of care from pediatric to adult services or providers. Beyond patients, studies of caregivers and/or clinicians of patients who met these criteria were also eligible. When consensus could not be reached, a third author (MT) was consulted. The included studies did not lend themselves to critical appraisal.

### Provider experience

Following the review of the literature, an audit was conducted of colorectal services listed on the ONE in 5000 Foundation website [23]. The ONE in 5000 Foundation provides awareness, information, and support for those born with an anorectal malformation (ARM), a condition that is largely surgical in management. The Foundation has invested significant time in compiling a global list of hospitals that provide colorectal care. While not an exhaustive list of Centers managing chronic constipation it is believed that this list represents those providing surgical management of colorectal conditions, including chronic constipation. In the absence of a similar list of leaders in the medical management of chronic constipation and the surgical nature of the identified literature, this methodology enabled sampling of those who care for pediatric patients with more severe constipation who might be expected to require transfer to adult health services.

Each of these hospital’s websites was reviewed to ascertain which facilities described specialized Colorectal Centers or programs. These specialized Colorectal Centers were invited to share their experience of managing patients with chronic functional constipation, particularly during adolescence and in preparing for transfer to adult providers. An initial email was sent inviting them to engage in a brief survey, followed by a reminder email one month later. This survey asked the following four, largely open-ended questions:

(1) Where are patients with chronic constipation treated in your service (e.g., general medicine, pediatrics, colorectal team, gastroenterology team)?

(2) Do you continue to treat patients with chronic constipation into adolescence and young adulthood?

(3) Where do you transfer these patients to?

(4) Do you have a policy or guidelines to support the transition of adolescents with chronic constipation to adult providers? If so, would you be happy to share them with us?

Respondents were given the opportunity to view a draft of this paper and asked to indicate any concerns with the material included prior to submission.

## Results

**Fig. 1:**
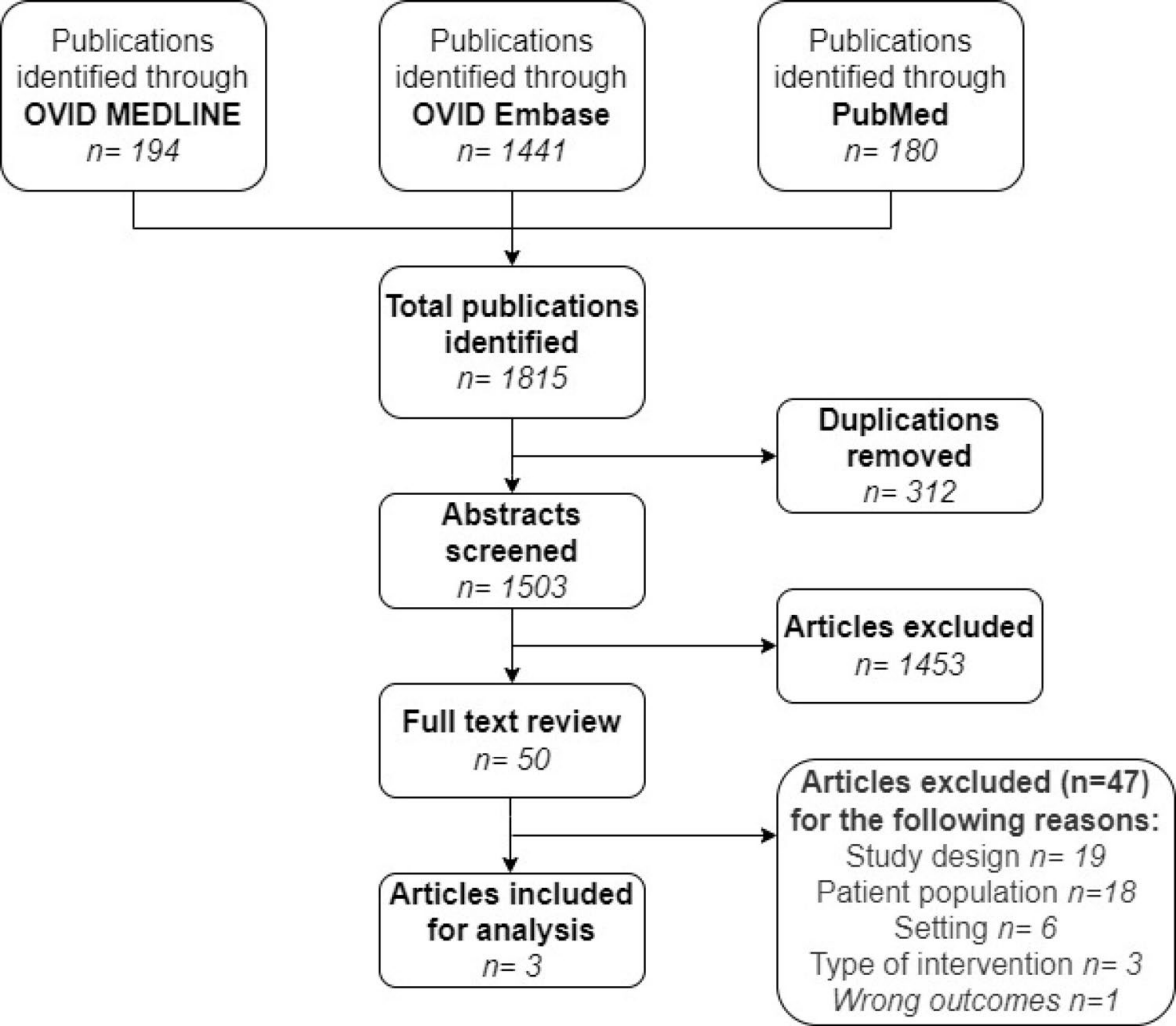
Study selection following the PRISMA guidelines

### Literature review

The initial systematic search identified 1815 titles, from which 1503 abstracts were screened, and 50 full text papers were reviewed. This resulted in three papers which met the search criteria (see Fig. 1 and Table 1). Each of these studies was conducted in high resource countries, had been published in the last five years and focused on the management of chronic constipation by surgical teams. There were no papers identified that focused on transitional care of patients with chronic functional constipation who were managed by medical teams. One paper described a multidisciplinary unit in the USA, the roles of clinicians and the points of patient contact [24]. The second paper was a retrospective review of records of children who received a Malone antegrade continence enema (MACE) for management of ARM, Hirschsprung Disease (HD) and chronic functional constipation. This study described the portion of patients who did not require ongoing specialist care and those who had a need for transition to adult providers. The author noted a portion of the three subgroups required ongoing care by adult providers, with chronic constipation having similar rates of ongoing need to the HD cohort [25]. The third paper described a prospective cohort study that sought to better understand the role of the antegrade continence enema (ACE) in the long-term management of constipation [26].

**Table 1:** Characteristics of included studies

First author (Year)	Study Aims	Data Collection	Population description	Participants (*n*) & response rate (%)	Age	Condition
Vilanova-Sanchez, A. (2019)	To describe the process of creating a collaborative process for the care of complex colorectal patients.	Description of the roles of the clinicians working within the MDT and the types and frequencies of interactions with patients.	31 staff − 3 ped. colorectal surgeons, 16 nurses, 2 admin, 2 social workers, 1 research assistant, 2 child life specialists, 1 psychologist, 2 clinical fellows and 1 research fellow. 1258 patients.	31 staff and 1258 patients. Response rate- N/A.	Patient age not provided. Pediatric setting.	321 with severe FC (26%).
Peeraully, R. (2022)	To analyze the outcomes and follow up of children who underwent the MACE procedure in a UK tertiary paediatric surgery unit and to assess the longer-term experience of the unit in managing children with MACE especially those entering adolescence and needing transitioning of care to adult services.	Retrospective cross-sectional analysis of patient files.	Patients with MACE inserted between 1998–2020 in a UK tertiary pediatric hospital for management of CIC, ARM or HD.	*n* = 95 Response rate- N/A.	Mean age at surgery 9.4 years (3–19 years).	59 (62%) with CIC.
Keshtgar, A. (2022)	To determine the long-term outcome of the ACE stoma for treatment of chronic constipation and soiling in children.	Prospective review and follow-up of clinic patients. Cohort study.	Children who underwent formation of ACE stoma for treatment of chronic constipation and fecal incontinence at the Evelina London Children’s Hospital from September 2008 to October 2020.	58 patients. Response rate- unknown.	Median age at operation 11 years (range 4–15 years).	50 (86%) had FC.

### Provider review

The ONE in 5000 Foundation website listed 154 websites (http://www.onein5000foundation.org). Two of these were unable to be accessed online, which left 152 websites, of which 27 described some form of specialized Colorectal Center. Each of these 27 Centers was emailed, although one email was unable to be delivered. Of the 26 Centers we were able to contact, responses were obtained from 15 (57.7%) Centers. Most were located in the USA (11), with two Centers in Australia and one each in Canada and Sweden.

### Services that manage chronic constipation

The three included papers all focused on the care of constipation in tertiary hospital settings. In two of the studies, the patients represented the most extreme severity of constipation and were managed by surgical teams as was apparent from the presence of indwelling devices [25, 26]. The third study analyzed the role of a specialized multidisciplinary unit that was specifically designed to manage complex colorectal conditions, including severe functional constipation [24].

Responses from the 15 Colorectal Centers suggested that patients with chronic functional constipation are managed by a variety of clinical teams in the tertiary setting, including general medicine, pediatrics, gastroenterology, motility specialists, bowel management services and, in many cases, colorectal surgical teams (Table 2).

**Table 2:** Summary of personal correspondence

Hospital	Who manages CC?	Do you treat patients up to the age of 18 years for CC?	Where do you transfer patients to?	Do you have a specific CC transition policy?
C.S. Mott Children’s Hospital, Ann Arbor, USA	GI	Yes	Adult BMP	No
Children’s Mercy, Kansas City, USA	CR, GI, GM, PED, SUR	Yes	Adult GI or adult SUR	No
Children’s National Hospital, Washington D.C., USA	GI, MOT, SUR	Yes	GI (Pediatric or adult depending on age)	No
Cincinnati Children’s Hospital, Cincinnati, USA	GI, GM, PED, SUR	Yes	No referral points at present	No
Karolinska University Hospital, Stockholm, Sweden	GI, GP, PED, SUR	Yes	Adult neuro-gastroenterology	No
Mass General Hospital for Children, Boston, USA	GI, MOT, PED	Yes	Adult clinicians at Mass General	No
Montreal Children’s Hospital, Montreal, Canada	GI, PED, SUR	Yes	Adult GI if diagnosis of dysmotility, otherwise PCP	No
Nationwide Children’s Hospital, Columbus, USA	GI, GM, PED, SUR	Yes	The Ohio State University Wexner Medical Center	Yes
Nicklaus Children’s Hospital, Miami, USA	GI, SUR	Yes	Continue to treat at Nicklaus	No
Pheonix Children’s Hospital, Phoenix, USA	BMP, GI, MOT	Yes	Adult GI	No
Primary Children’s Hospital, Salt Lake City, USA	CR, GI, PED	Yes	Adult GI or GP	No
Seattle Children’s Hospital, Seattle, USA	CR, GI, MOT	Yes	MOT	No
Shriners Children’s/University of California, Davis, Sacramento, USA	GI, MOT, SUR	Yes	Adult GP, adult GI, adult SUR.	No
The Children’s Hospital at Westmead, Sydney, Australia	CR, GI, GM, PED	No	Use Trapeze transition service	No
The Royal Children’s Hospital, Melbourne, Australia	CR, GI, GM, PED	Yes	Adult CR	No

### Timing of transition planning

There was significant variability in the timing of transition planning. The literature indicated that in some tertiary settings transition planning commenced between 14 and 16 years of age [25, 26]. The most common target age for transfer was 18 years [26]; however, some transferred to adult providers around 16 years of age [25]. There was also significant variability in the age of transfer within the same Center. For example, while Peeraully and colleagues reported a mean age of transfer at 18.9 years, this ranged from 16.7 to 24.1 years [25].

Similarly, correspondence with the Colorectal Centers suggested that transition planning commenced as early as 12 years of age in some settings. The most common target age for transfer was reported as 18 years; however, consistent with the reviewed papers, some transferred to adult providers around 16 years of age.

### Disciplinary responsibility for transition to adult healthcare

The limited data available in the literature suggested that the responsibility of transition to adult health care for adolescents with surgically managed chronic functional constipation sat largely with surgeons. However, there was variability in approaches from one paper to the next. Vilanova-Sanchez and colleagues described the value of working with a colorectal surgeon who is registered to practice in both pediatric and adult surgery settings [24]. This model enabled the surgeon to carefully oversee surgical care as patients transferred from pediatric to adult services. This contemporary model contrasts with the more traditional approach reported by Keshtgar and colleagues where adolescent patients were described as attending outpatient appointments with both an adult and pediatric surgeon from 14 years of age [26]. While Keshtgar and colleagues did not clearly identify who was responsible for transitional care, the model inferred shared responsibility in supporting adolescent patients until the age of 18 years when transfer to adult services occurred [26].

Interestingly, while correspondence with providers did not specifically address who was responsible for transition, the unique model of dual qualified surgeons working across both pediatric and adult settings was also evident. One Center reported this approach to supporting patients through the period of transition.

### Transitional care programs

There were no transitional care programs identified within the literature review. Two of the 15 Colorectal Centers revealed they use external transitional care programs, such as Got Transition^®^ and Trapeze, to support the transition of adolescent patients [27, 28]. Got Transition^®^ is an American resource that supports adolescents and young adults (ages 12 to 23 years), as well as their clinicians and families, through providing education about the healthcare transition. The program consists of six pillars commencing with the introduction of a transition policy within a service, thorough planning, readiness and transfer, before concluding once the young adult is established with their adult provider. Trapeze is an Australian program, based at the Sydney Children’s Hospitals Network. Trapeze differs in that they have a group of multidisciplinary staff who are trained to provide direct support to adolescents and young adults though the transition period. Both programs provide comprehensive transitional support, although while Got Transition^®^ aims to empower clinicians to support adolescents through transition, Trapeze has specialized staff who work in addition to the regular care team.

### Transitional care policy

While none of the three papers described the role of transitional care guidelines, Vilanova-Sanchez and colleagues reported that a defined transition pathway facilitated support of adult clinicians who had received referrals for new patients presenting with congenital issues [24].

One Center reported the use of generalised transitional care guidelines to provide a timeline and checklist for all adolescent patients, including those with constipation, as they move through the transition period. Other Centers reported the value of service-wide transitional care programs that were available for all adolescent patients but did not report their use of such programs. They also acknowledged that there would be value in having transitional care programs more specifically oriented to adolescents with chronic functional constipation. Two Centers, one in the USA and the other in Australia, reported current efforts to develop such specific transitional care programs. Conversely, most providers reported that they did not use any professional association guidelines or hospital policy to inform their approach to transition to adult health care for patients with chronic functional constipation. In some cases, this was reportedly due to small numbers of adolescents or challenges with geographical boundaries and insurance coverage, which facilitated the need for individualized transitional care plans.

The most comprehensive transitional care program for adolescents with surgically managed chronic functional constipation appeared to be based at Nationwide Children’s Hospital, Columbus, USA. From the age of 12 years, it was reported that their patients complete annual transitional readiness surveys which identify areas for intervention in preparation for transfer to an adult provider. From the age of 16 years, social workers further support transitional readiness. When the multidisciplinary team is confident that a patient is stable from a medical perspective, and prepared for transfer to an adult provider, the patient is transferred to a dual-qualified pediatric and adult surgeon at the affiliated adult hospital (A. Gasior, personal communication, 18th Feb 2024).

### Type of adult provider

Patients are transferred to a variety of providers and clinicians according to ongoing care needs and available referral pathways. According to the literature, adolescents with indwelling devices, such as antegrade continence enemas, were most commonly transferred to adult colorectal surgeons, although this did not preclude concurrent use of community providers such as pelvic floor services [24, 25]. Peeraully and colleagues also noted that transfer of care was often more challenging for patients with chronic functional constipation who had not undergone bowel surgery [25].

The contacted Centers detailed a myriad of referral options for patients with chronic functional constipation. Consistent with the literature, providers generally referred patients with indwelling devices to adult colorectal surgeons. In contrast, adolescents who were managing their constipation without an indwelling device were more generally referred to community providers, such as general practitioners, bowel management clinics, pelvic floor services, and other nurse-led clinics. For patients whose constipation was poorly managed but who did not have an indwelling device, referral pathways included gastroenterology units, colorectal surgical units, hospital-based motility clinics, or bowel management programs. In some cases, intrahospital transfer occurred. Within pediatric hospitals, pediatric colorectal surgeons sometimes transferred patients to pediatric gastroenterologists who subsequently transferred adolescents to adult services at a later stage. In general hospitals that manage patients across the life course, transfer typically occurred from pediatric to adult providers within the same institution.

### Barriers and enablers of quality transitional healthcare

A number of barriers and enablers of transition were identified in the literature. A trusting relationship with pediatric providers was found to be a barrier to successful transition to adult providers; some adolescents described feeling abandoned in the transfer process [25, 26]. However, good communication between pediatric and adult providers was also found to enable successful transfer [25]. Good communication could facilitate agreement between pediatric and adult providers and was reported to enable the sharing of expertise for better patient care [26].

The availability of qualified and experienced adult clinicians was found to impact on the success of transition, both positively and negatively. Where there were suitably qualified clinicians, transition was more likely to be successful than in areas that did not have access to such experience [26]. This was particularly problematic for adolescents and young adults who had an indwelling device for the management of their constipation, as it was apparent from the literature that not all adult surgeons were familiar with these devices [26]. A powerful enabler identified in the literature reviewed was the involvement of dual-qualified surgeons who were working across pediatric and adult surgical spaces and were therefore able to support adolescents and young adults through the period of transition [24].

Vilanova-Sanchez and colleagues suggested that the relatively small number of young adults requiring ongoing care meant that service reorganization into fewer, specialized units may be a strategy to improve outcomes in this cohort [24]. This is consistent with the paper by Peeraully and colleagues that also suggested that case numbers meant it was not feasible to develop specialist Centers in all regions [25].

Commencing transition planning early was considered to enable more successful transition of care [24, 26]. Conversely, inadequate long-term follow-up was found to reduce the likelihood of successful transition [25]. Keshtgar and colleagues also suggested that delivering more standardized transitional care across all specialties would help improve transitional care outcomes for a wider group of adolescents than just those with chronic functional constipation [26]. The provision of developmentally and psychosocially appropriate care that was continuous and coordinated was reported to improve the likelihood of successful transition [26]. In each of the three papers in this review, reference was made to the impact of psychosocial stressors and the importance of a multi-disciplinary team that could provide psychological support [24–26].

The Centers contacted were not asked to identify barriers or enablers to the successful transition of care for patients with chronic functional constipation.

## Discussion

Given the prevalence of constipation in childhood and the impact of this on adolescents, a striking finding from this systematic review was that only three studies were identified that reported any aspect of transition to adult health care. Each of these three papers emanated from surgical programs, as did the 15 specialist Centers we surveyed, most of which were primarily developed to manage children and adolescents with anorectal malformations (ARM) and Hirschsprung disease (HD). In comparison to ARM and HD, where management follows a clearly defined pathway after surgical intervention in early childhood, constipation in childhood is highly variable in its severity, response to treatment and impact. This has resulted in a diversity of treatment pathways and specialist involvement, which make it difficult to estimate the proportion of children who will require transfer to adult services due to relapsing or intractable problems. A longitudinal study of 418 children (average age 8 years) managed by a single site motility service in the Netherlands suggested that 60% of children were successfully treated at one year and 80% by eight years. However, in those aged 16 years or older, constipation remained present in 30%, with the majority receiving no active treatment or follow-up [7]. Unlike ARM and HD, where the need for long-term follow-up and eventual transfer is anticipated from the outset, constipation may not be perceived as a condition warranting formal transition pathways, despite evidence that a significant proportion of patients continue to experience symptoms into adulthood.

Our audit of 15 specialist Colorectal Centers revealed significant variability in transition processes. Notably, all 15 Centers were academic programs, and findings may therefore not be representative of the broader landscape of colorectal care providers. In the 15 included Centers, there was no consensus on the timing of commencement of transition planning, nor to whom patients are transferred, even within this largely surgical sample. The only common feature was that adolescents with indwelling devices required tertiary care. However, several pediatric providers reported challenges identifying adult surgeons who were willing to assume the ongoing care for indwelling devices, which may reflect that current expertise is largely confined to the pediatric setting. Unfamiliarity with devices such as the antegrade continence enema (ACE) among adult colorectal surgeons has been identified as a specific barrier, and while targeted education may address knowledge gaps, this alone is unlikely to be sufficient. Adult colorectal surgery is heavily oriented toward oncological and inflammatory bowel disease management, and the functional and congenital conditions that dominate pediatric colorectal practice occupy a comparatively marginal role in adult training and scope of practice.

As the provision of transitional care becomes more common in pediatric settings, general transitional care programs have been developed that support the process of transition to adult healthcare for patients with a variety of conditions. We found no evidence that these programs, largely based on the principles of caring for adolescents with complex medical conditions, did not also suit the needs of adolescents with chronic functional constipation [29–31]. Indeed, consistent with these programs, each of the three papers on adolescents with chronic functional constipation found that high-quality communication between pediatric and adult providers was fundamental to successful transition, as was early commencement of transition planning, care co-ordination, access to a multidisciplinary team and careful consideration of the psychosocial implications of chronic constipation [24–26]. Paradoxically, positive relationships with pediatric providers was identified as a barrier to successful transition. As with other conditions, this most likely reflects that without appropriate transition planning, many adolescents and their families will struggle to leave familiar environments where trust and rapport had been established with pediatric providers over a prolonged period of time [25, 26].

This review found that one of the challenges of successfully transferring adolescents with chronic functional constipation was the small number of skilled adult clinicians. Dual qualified surgeons who can traverse pediatric and adult settings and contexts were shown to be especially valuable, although very few settings have access to this resource. While this model provides high-level continuity of care as adolescents transfer from pediatric to adult care, it is unlikely to be widely scalable. In this regard, a trade-off may be required between delivery of care close to home versus access to more specialized services. Alternatively, specialist nurses are often at the forefront of bowel management programs and may be better placed to provide tailored and scalable transitional care.

The identified literature was all surgical in nature, indicating that while there is potentially work happening in the medical setting for transition of patients with chronic constipation, there remains a gap in the published literature. Additionally, given our surgical setting, and the fact that the only published transition literature is surgical based, advice was sought from those managing chronic constipation in surgical settings. Subsequently, the findings of this paper are limited to patients with chronic constipation who are managed in surgical settings. While a limitation of the current study, it is reasonable to assume that those with more severe presentations of chronic constipation will be similarly managed by surgical teams at some point in their pediatric care. Little was found to describe the care needs of adolescents and young adults with less severe disease who are often managed in a variety of primary care settings. Such diversity of care settings suggests there will be an underestimate of the prevalence of chronic functional constipation within the adolescent population from hospital settings. At the very least it indicates the need for greater understanding of chronic functional constipation, its treatment and how best to support these patients across the life course, including their transition to adult services if required. Although the broad principles of transition are likely applicable across both medically and surgically managed patients with chronic functional constipation, the complexity of transfer is greatest for those with indwelling devices, where continuity of care is contingent on identifying adult surgeons with experience in what are currently largely pediatric-specific procedures. While this review only captured patients managed in surgical settings, it highlights that at least a portion of patients with chronic functional constipation require transfer to adult care and the important role that surgeons play in this process.

## Conclusion

Successful management of chronic functional constipation represents challenges to pediatric and adult clinicians alike. There remains a need to better understand the transitional care requirements of adolescents with chronic functional constipation, which would be aided by greater clarity about what proportion of children, both managed in medical and surgical settings, are predicted to require ongoing care in young adulthood, whether in primary or tertiary services.

## Supplementary Information

Below is the link to the electronic supplementary material.

Supplementary Material 1

## Data Availability

No datasets were generated or analysed during the current study.

## Abbreviations

ARM: Anorectal malformation
ACE: Antegrade colonic enema
BMP: Bowel Management Program
CC: Chronic constipation
CIC: Chronicidiopathic constipation
CR: Colorectal
FC: Functional constipation
GI: Gastroenterology
GM: General Medicine
GP: General practitioner
HD: Hirschsprung disease
MACE: Malone Antegrade Continence Enema
MDT: Multidisciplinary team
MOT: Motility Clinic
PCP: Primary care provider
PED: Pediatrician
SUR: Surgical
