# Supplementary material for: Transition from pediatric to adult healthcare for patients with chronic functional constipation: a scoping review and provider insight
Source: Pediatr Surg Int. 2026 Jul 20;42(1):314. doi: 10.1007/s00383-026-06537-w (PMC13385162; doi:10.1007/s00383-026-06537-w)
Supplement: Supplementary file 1 — Supplementary Material 1 [file 383_2026_6537_MOESM1_ESM.pdf]

SUPPLEMENTARY MATERIAL

Transition from paediatric to adult healthcare for patients with chronic functional constipation: a scoping review and provider insight

PEDIATRIC SURGERY INTERNATIONAL

Emma J. Moore [1,2], Melissa Y. Tien [1], Susan M. Sawyer [1,2,3], Sebastian K. King [1,2,4], Misel Trajanovska [1,2,4]

[1] Murdoch Children's Research Institute, 50 Flemington Road, Melbourne, Victoria, Australia

[2] Department of Paediatrics, University of Melbourne, 50 Flemington Road, Melbourne, Victoria, Australia

[3] Centre for Adolescent Health, The Royal Children's Hospital, 50 Flemington Road, Melbourne, Victoria, Australia

[4] Department of Paediatric Surgery, The Royal Children's Hospital, 50 Flemington Road, Melbourne, Victoria, Australia

Corresponding author: Ms Emma Moore, Murdoch Children's Research Institute, 50 Flemington Road, Parkville, Melbourne Victoria, Australia 3052, [emma.moore97@mcri.edu.au].

Supplementary Material 1: Search Strategy

Medline

1. exp constipation/
2. (Colonic-inertia or constipation or dyschezia or obstipation).tw,kf.
3. 1 or 2
4. "continuity of patient care"/ or transition to adult care/
5. Transitional Care/
6. transition*.tw,kf.
7. 4 or 5 or 6

8. 3 and 7
9. limit 8 to (comment or editorial or letter)
10. 8 not 9
11. limit 10 to (english language and yr="2011 -Current")

Embase

1. exp constipation/
2. (Colonic-inertia or constipation or dyschezia or obstipation).tw,kf,dq.
3. 1 or 2
4. patient care/ or "bladder and bowel management"/
5. transition to adult care/
6. transitional care/
7. transition*.tw,kf,dq.
8. 4 or 5 or 6 or 7
9. 3 and 8
10. limit 9 to (conference abstract or conference paper or "conference review" or editorial or letter)
11. 9 not 10
12. limit 11 to (english language and yr="2011 -Current")

PubMed

#1 Title/Abstract

“Colonic-inertia” OR “constipation” OR “dyschezia” OR “obstipation”

#2 Title/Abstract

“patient-care” OR “transition*” OR “transitional-care”

#3 All fields

NOTNLM OR publisher[sb] OR inprocess[sb] OR pubmednotmedline[sb] OR indatareview[sb] OR pubstatusaheadofprint

#4 #1 AND #2 AND #3

#5 #1 AND #2 AND #3 Filters: English

#6 #1 AND #2 AND #3 Filters: English, from 2011 - 3000/12/12

#7 #1 AND #2 AND #3 Filters: Books and Documents, English, from 2011 - 3000/12/12

#8 #1 AND #2 AND #3 Filters: Books and Documents, Editorial, English, from 2011 - 3000/12/12

#9 #1 AND #2 AND #3 Filters: Books and Documents, Editorial, Letter, English, from 2011 - 3000/12/12

#10 #1 AND #2 AND #3 Filters: Books and Documents, Editorial, Lecture, Letter, English, from 2011 - 3000/12/12

#11 #6 NOT #10
